# A morphometric study of normal and varus knees

**DOI:** 10.1007/s00167-014-3337-2

**Published:** 2014-09-27

**Authors:** Pramod Kumar Puthumanapully, Simon J. Harris, Anthony Leong, Justin P. Cobb, Andrew A. Amis, Jonathan Jeffers

**Affiliations:** 1 Department of Mechanical Engineering, City and Guilds Building, Imperial College London, London, SW7 2AZ UK; 2 Department of Surgery and Cancer, Charing Cross Hospital, Imperial College London, London, W6 8RF UK

**Keywords:** Morphometry, Varus knee, Morphology, Rotational alignment, Axes

## Abstract

**Purpose:**

The aim of the study was to investigate varus and normal knee morphologies to identify differences that may affect knee replacement alignment or design for varus knees.

**Methods:**

Computed tomography scans of varus and normal knees were analyzed, and geometric shapes, points and axes were fit to the femur and tibia independently. These points were then projected in the three anatomical planes to measure the variations between the two groups.

**Results:**

In the femur, varus knees had less femoral anteversion (*p* < 0.0001) and a larger medial extension facet (*p* < 0.05) compared with normal knees. In the tibia, the tubercle was found to be externally rotated in varus knees (12°), with a significant increase in the coronal slope (*p* = 0.001) and the extension facet angle (*p* = 0.002).

**Conclusions:**

The study highlighted the differences and similarities found between the two groups, which raises awareness on changes required during surgical intervention and component placement or design for a varus knee. This is particularly relevant for the design of patient-specific instrumentation and implants.

**Levels of evidence:**

Diagnostic study, Level III.

## Introduction

Varus alignment has been shown to increase the risk of osteoarthritis (OA), especially in the medial condyle [33], and also accelerate disease progression in knees with existing OA [5]. Due to the varus alignment, the knee experiences higher loads through a larger adduction moment during gait, that increases loading through the medial condyle and reduces loads passing through the lateral condyle [1], thus causing or exacerbating the problems of OA on the medial condyle.

The biomechanics of the knee is governed largely by the shape of the distal femur and proximal tibia, and the relationship that exists between their articulating surfaces. A normal knee, defined as having a neutral alignment, has a limb angle of 180° ± 3° [23], measured between the mechanical axis of the femur and the mechanical axis of the tibia. While the morphology and angulation of normal knees are well defined in the literature [13, 35, 36], varus knees have been primarily classified based on the alignment of the femur and tibia in just the coronal plane, with a mechanical tibiofemoral angle of 177° or lower. Varus and valgus knees have been compared, but limited to a comparison of the flexion facets [18] without incorporating the extension facets, which are most affected in medial OA. In addition, there are very few studies characterizing the variations in local morphology and alignment of varus knees [9] and specifically comparing their differences with normal knees [18, 25].

Condylar morphometry, which involves the study of the geometry and related axes and their associations, can be used to investigate features of the condyles and their relative movements [11, 12]. Rotational alignment, which is critical to the kinematics and contact pressures of the implanted knee in total knee replacement (TKR), is often defined using axes and landmarks whose associations with each other are well understood. For example, the transepicondylar axis and the posterior condylar axis of the femur are often used to align the femoral component of knee prostheses [30, 36] and the posterior condylar axis of the tibia, the condylar centers axis and the tibial tubercle axis used to align tibial components [7]. There is a risk that axes used to align components may change with the varus deformity, leading to implant misalignment and adverse biomechanics of the replaced joint. This is particularly timely given the recent heavy marketing of patient-specific instrumentation by manufacturers and the risk that such guides may align implants to the wrong place in varus knees if they are based on the wrong reference features.

It was hypothesized that the axes and features used for alignment or patient-specific implant design are different between normal and varus knees, and recognizing and identifying these can help improve alignment of components, and patient-specific instrument and implant designs for a varus knee. To test this hypothesis, established geometrical features and axes of normal and varus knees were measured using a computed tomography (CT) protocol and any significant differences investigated.

## Materials and methods

A total of 56 knees, 31 varus (173° ± 2.4°) and 25 normal (181.5° ± 1°), were CT scanned using a Siemens Sensation 4 four-slice CT scanner, following the imperial knee protocol for low-dosage tomography that also scans the femoral head/neck and ankle to allow mechanical axes to be resolved [16]. Of the 56 knees, for the measurements on the tibia, only 29 varus and 18 normal tibiae could be included due to insufficient CT slices to resolve some of the key axes. The varus knee group had OA, but this was limited to the anterior of the medial femoral/tibial condyle. To maintain consistency across the entire dataset, a protocol of landmark identification and measurements was developed and followed in each case. In-house CT software was used to threshold the grayscale values and allow visualization of the joint surfaces. The femur and tibia were analyzed separately as the relative alignment between femur and tibia was not considered a valid measure because of the supine unloaded body position during the CT scan. The methods employed for the femurs and tibias are described below.

### Measurements of the femur

**Fig. 1:**
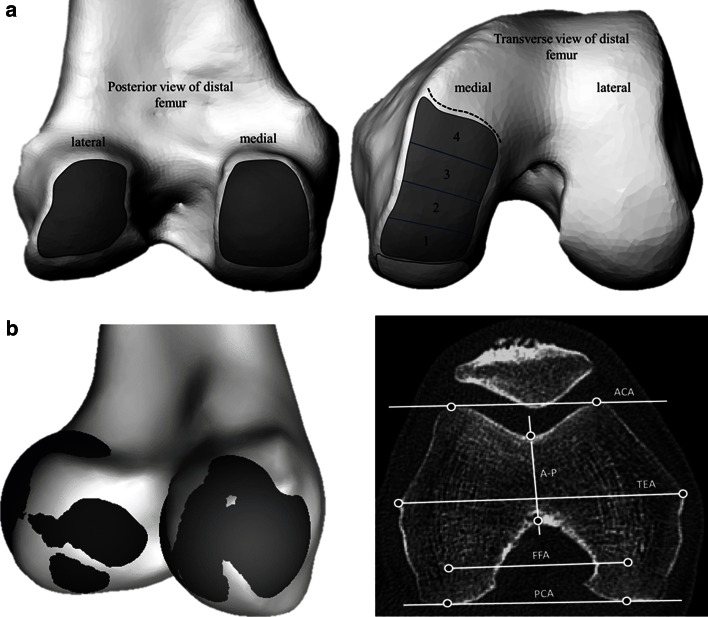
**a** Regions used to define and place markers on the flexion facets (*left*) and extension facets (*right*) in the distal femur to fit spheres. Boundaries of the facets were identified on CT scans; the extension facet extended until the medial sulcus terminalis (*dotted line*) and was split into four sections for more detailed analysis. **b** Visualization of the spheres fit to the flexion facets of the medial and lateral condyles, and transverse CT slice illustrating commonly defined axes for the distal femur (*right*)

The femur was first orientated to a tri-spherical coordinate system formed by the centers of spheres fit to the femoral head and the medial and lateral flexion facets (FFs) of the knee. The mechanical axis of the femur (MAf) was identified as the line connecting the center of the femoral head and the inter-condylar notch, defined in this study as the most distal center of the notch. Landmarks were then identified to define the following axes—anatomical transepicondylar axis (TEA), femoral posterior condylar axis (PCAf), anterior condylar axis (ACA) and Whiteside’s line (AP) (Fig. 1). The femoral neck version was also measured with respect to the PCA. The extension facet (EF) of the medial condyle was defined as bounded anteriorly by the sulcus and posteriorly by projecting inferiorly the posterior edge of the depression associated with the origin of the medial gastrocnemius tendon. Spheres were defined to represent the flexion facet (FF) surfaces of the medial and lateral condyles and their centers used to define the flexion facet axis (FFA, Fig. 1). The extension facet was split into four equally spaced areas from posterior to anterior and spheres fit to each of these areas to investigate local changes in sphericity. The extension facet on the lateral condyle of both varus and normal knees was not measured owing to its flatter surface and unreliable sphere fitting as has been commented on previously [19].

For fitting the sphere on to the condylar surfaces, over 50 points were used to fit spheres of best fit for the regions defined. The coordinates of all the landmarks, including sphere centers, were then projected in the coronal, sagittal and transverse planes relative to the tri-spherical axis defined previously, and measurements were carried out in each of the planes using a custom written code in MATLAB (MathWorks, MA, USA). The diameters of the spheres, the angles formed between the various axes, point-to-point distances and point-to-axes distances were calculated in each of the anatomical reference planes. To eliminate problems related to the size variation in the groups, distances were normalized to the length of the TEA for statistical analyses. Considering that the focus was on the distal femur, the TEA was thought to be the most appropriate.

### Measurements of the tibia

**Fig. 2:**
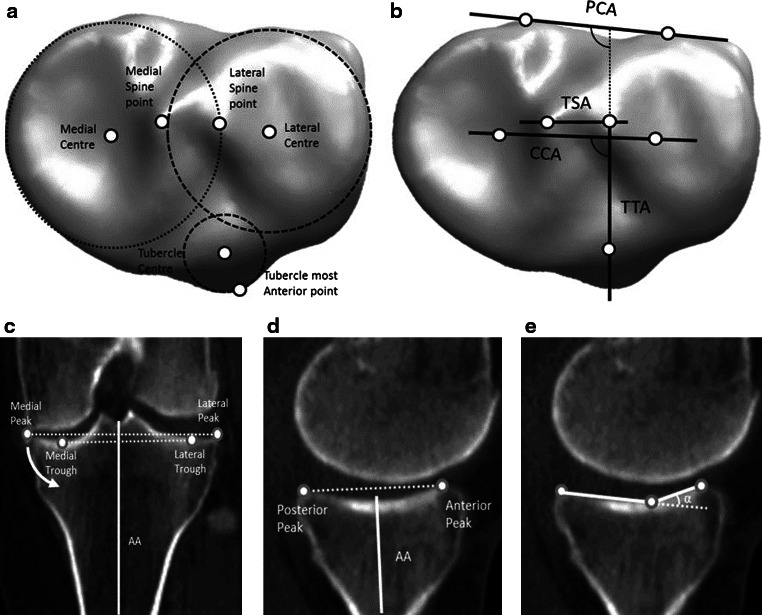
**a** Transverse view of circles and their centers fit to the medial and lateral plateaus, along with the tuberosity. **b** The axes defined for the tibia in the transverse plane. **c** Points used for measuring the coronal slope with the anatomical axis (superior: peaks and inferior: troughs) with the *arrow* indicating direction of increasing slope **d** the medial AP slope with the mechanical axis and **e** α, the medial extension facet angle

The tibia was orientated to axes that were defined by the centers of the circles fit to the medial and lateral plateaus in the transverse plane and the center of the talus. To avoid erroneous measurement due to the presence of osteophytes, the circles were fit in a plane, 5 mm distal from the base of the medial inter-condylar spine. A circle was also fit to the tibial tubercle in the transverse plane and the center marked. The mechanical axis (MAt) was defined as the line connecting the center of the tibia to the talus, with the proximal anatomical axis (AA) defined as the line bisecting the proximal medullary canal. Landmarks were then located to define the axes used by Cobb et al. [7]: posterior condylar axis of the tibia (PCAt), condylar centers axis (CCA) and the tibial tubercle axis (TTA) (Fig. 2a).

The following landmarks were also identified: the peaks of the medial and lateral spines; the most anterior point of the tibial tubercle; the most superior (peaks) and inferior points of the tibial condyles in the coronal plane and sagittal planes to define the coronal and AP slopes; and the points defining the extension facet angle in the sagittal plane (Fig. 2c–e). The coronal slope was measured as the angle between the tibial plateau and the normal to the anatomical axis. These points were then projected onto the anatomical reference frames relative to the previously defined functional axis and measurements carried out as described for the femur.

### Statistical analysis

The sample size used in the study was in keeping with previous studies that have investigated morphometry [24, 25]. A complete set of measurements for each knee was completed and repeated by the lead author after a period of 3 weeks, and the intraclass correlation coefficient was used to determine the intraobserver reliability. For comparison between varus and normal groups, statistical analyses were carried out in SPSS (version 21, IBM, Chicago, IL, USA) using two-sample *t* tests or Mann–Whitney tests depending on the normality of the data, determined using the Shapiro–Wilk test. The significance set at *p* < 0.05 with an additional Bonferonni correction applied for groups of related measurements.

## Results

The reliability of the measurements was found to be excellent on the femur for both varus and normal knees; the intraclass correlation coefficient was found to be between 0.85 and 0.96 for all of the landmarks identified. In the tibia, there was very good agreement for the majority of the landmarks (0.87–0.93) except for landmarks used for the coronal slopes (0.72) due to the presence of osteophytes in eight of the varus tibiae.

### Femoral condylar geometry and rotational axes

A significant difference was not found between the two groups for the diameters of the FF on the medial or lateral condyles. However, the EF on the medial condyles was found to be significantly larger in varus knees; all of the individual sections analyzed were significantly larger (*p* < 0.05) than their counterparts in the normal group with mean differences ranging between 8 and 12 mm for individual sections. The diameters of the fit spheres for the flexion and extension facets are summarized in Table 1. The center points of the spheres fitted to the medial extension facet sections translated anteriorly from sections 1 to 4 by about 5 mm in both groups.

**Table 1:** Measurements made on the femur and tibia for varus and normal knees

Geometry	Varus (mm)	Normal (mm)
Femoral medial FF radius	20 ± 2	21 ± 2
Femoral lateral FF radius	21 ± 3	22 ± 3
Femoral EF medial section 1	35 ± 18*	25 ± 7*
Femoral EF medial section 2	36 ± 16*	25 ± 8*
Femoral EF medial section 3	37 ± 14 *	29 ± 10*
Femoral EF medial section 4	48 ± 23*	35 ± 15*
TEA length	81 ± 6	84 ± 8
PCA length	50 ± 5	52 ± 5
ACA length	37 ± 5	37 ± 4
FFA length	51 ± 5	54 ± 4
Tibial medial plateau radius	28 ± 3	27 ± 3
Tibial lateral plateau radius	23 ± 4	24 ± 3
Tibial tubercle radius	11 ± 2	11 ± 1
CCA length	25 ± 4	27 ± 4
TSA length	11 ± 2	12 ± 3

Femoral axis	PCA		A-P		FFA		ACA	
	Varus	Normal	Varus	Normal	Varus	Normal	Varus	Normal
TEA	7* ± 2	7* ± 2	2* ± 5	3* ± 6	7* ± 3	8* ± 3	9* ± 3*	11* ± 3*
PCA		5* ± 6	4* ± 6	2* ± 2	2* ± 2	2* ± 2*	4* ± 3*	
A-P			5* ± 7	5* ± 7	7* ± 5	7* ± 6		
FFA				3* ± 3	3* ± 3			

Tibial axis	CCA		TSA		TTA	
	Varus	Normal	Varus	Normal	Varus	Normal
PCA	8* ± 5	7* ± 2	12* ± 14	13* ± 9	108* ± 6**	96* ± 6**
CCA			11* ± 12	6* ± 8	100* ± 7**	89* ± 7**
TSA				99* ± 15**	84* ± 10**	

* *p* < 0.05, ** *p* < 0.001

The angles formed by the reference axes, TEA, PCA, FFA and AP did not differ significantly between the two groups. The results are also summarized in Table 1. The ACA was found to be significantly more in normal knees (~2°, *p* < 0.05) measured against the PCA and TEA.

### Local length changes and distance measurements

Significant differences were not found in the distances measured between the landmarks, or from landmarks to the defined axes.

### Femoral neck version

**Fig. 3:**
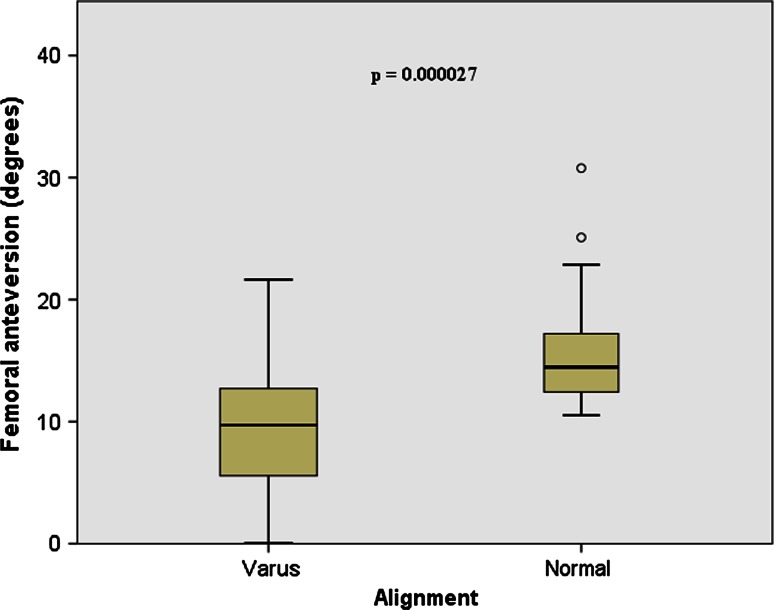
*Box* plot depicting the differences found between the varus and normal groups for femoral neck anteversion; varus femurs had less anteversion when compared to normal femurs

The femoral neck was significantly less anteverted for varus knees (9.4° ± 5°) compared with normal (15.7° ± 5°; *p* < 0.0001, Fig. 3). A weak positive correlation was found between varus angle and femoral neck version in the varus group.

### Tibial condylar geometry and rotational axes

The medial plateau was larger than the lateral plateau in both varus and normal tibiae, and no significant difference was found between the normal and varus groups for either medial or lateral plateau radii.

**Fig. 4:**
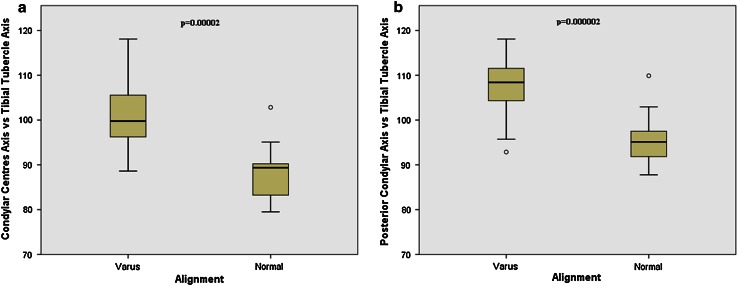
*Box* plot showing differences found between the two groups when the condylar centers axis and posterior condylar axis are measured against the tibial tubercle axis

The angle formed by the tibial PCA and CCA was not significantly different for varus and normal tibiae. The angles formed by the TTA and the tibial PCA were significantly larger (*p* < 0.0001) in varus tibiae, i.e., the tibial tubercle was rotated externally by 12° when compared to normal knees (Fig. 4).

### Medial extension facet angle and slopes

**Fig. 5:**
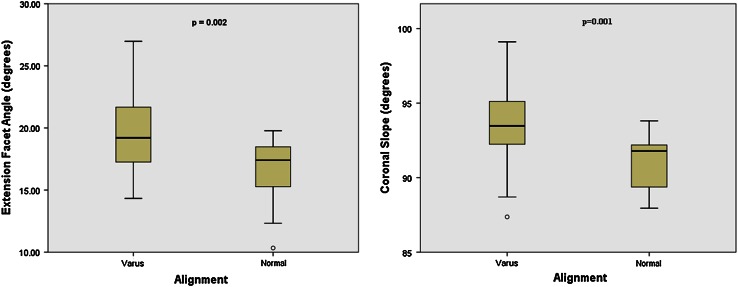
**a** *Box* plot showing differences between varus and normal tibiae for the extension facet angle and **b** the coronal slope

**Fig. 6:**
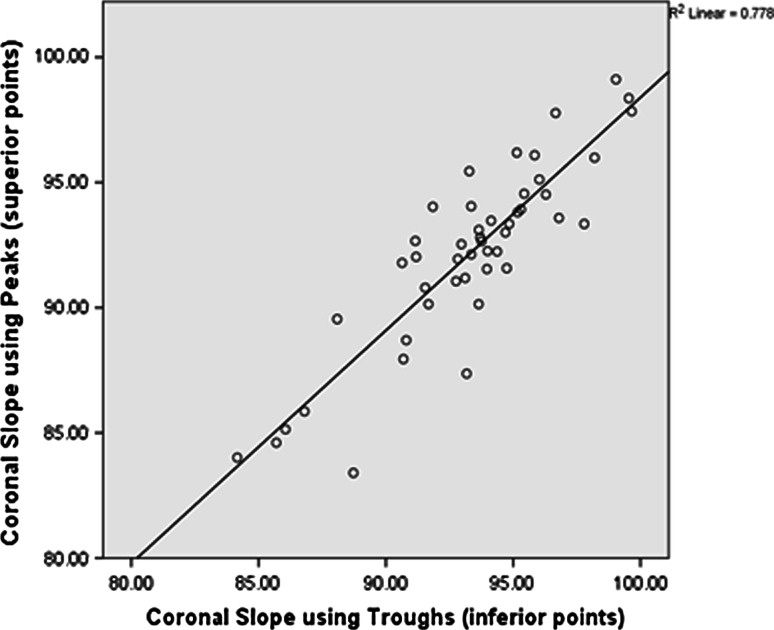
Scatterplots showing the strong positive correlation between coronal slope measured using the superior and inferior points

There was a small (2°) but significant increase in medial extension facet angle for the varus group (*p* = 0.002, Fig. 5a). The coronal slope was found to be significantly more (*p* = 0.001) in varus knees (3.5°) when compared to normal knees (0°) (Fig. 5b), indicating that the slope contributes to the varus deformity. Measuring the coronal slope of the tibia is often difficult due to the presence of osteophytes; therefore, it was investigated whether the coronal slope could be measured using the most inferior points (troughs) in the medial and lateral plateaus as opposed to the peaks (Fig. 2c). A strong correlation was found between the two methods of measuring the slope in the coronal plane (*r* ^2^ = 0.778; *p* < 0.0001; *n* = 47, see Fig. 6).

## Discussion

The major findings of this study were that, for varus knees, some reference axes and surface features are significantly different to normal knees. For the femur, there was less femoral anteversion in varus knees. In the tibia, the tubercle (and tibial tubercle axis) was externally rotated in varus knees and there was a medial tilt of the tibial plateau in the coronal plane. Axes were also identified that were not significantly different in varus and normal knees. On the femur these were the anatomical transepicondylar axis, posterior condylar axis and Whiteside’s line and, on the tibia, the posterior condylar axis and condylar center axis.

The presence of less femoral anteversion in varus knees when compared to normal knees is not surprising. Normal femoral version has been reported to be varied between 10° and 20° [6] with the clear evidence of increased anteversion causing internal rotation of the knee, which is closely associated with increasing valgus deformity of the knee [37]. Similarly, retroversion causes external rotation of the knee, is associated with varus deformity and has been shown to be closely related to the development of OA in adults [34]. The varus knee group in the study all presented with OA of the medial side, and this has also been shown to be related to external rotation of the knee [20]. Bretin et al. [4] showed that the tibiofemoral joint center of force moves medially with external femoral malrotation, and this has been further confirmed by finite element studies by Papaioannou et al. [31] who showed increased compressive forces in the medial compartment of the knee joint with decreasing anteversion. Thus, a statement on the predisposition of femurs with less femoral version angles and varus alignment leading to subsequent medial OA of the knee can be made.

Continuing with the femur, the study found the axes commonly used for femoral alignment did not differ between normal and varus knees. For both groups, the PCA was internally rotated by 7° with respect to the anatomical TEA, and Whiteside’s line had large standard deviations in its measurement which agrees with prior work [23, 26, 28].

For the tibia, the main finding was that the tibial tubercle, and consequently the tibial tubercle axis, was externally rotated in varus knees compared with normal knees. This is a common reference axis for axial alignment of the tibial tray in TKR [29], but it has been reported to be highly variable in different patients [29, 30], and, for this reason, concerns have been raised regarding its use for this purpose [7]. For the tibia, a small increase in extension facet angle, which engages the femur during extension (from 0° to 30°), was found. An increase in the extension facet angle has been attributed to the development of antero-medial OA of the knee [3, 21] which may be a causative factor in the increased risk of OA observed in varus knees in longitudinal studies [5, 33]. The coronal slope measured in varus knees demonstrated a slope toward the medial edge of the knee that may be a natural consequence of the varus deformity and excessive medial loading and erosion, which could be addressed by tibial osteotomy [2]. It is well known that the posterior tibial slope plays an important role in the kinematics of the natural and implanted knee and also influences joint laxity and ligament function [15, 17], and the results of the study were in agreement with previous work [27]. There was no significant difference found in the posterior tibial (AP) slope between normal and varus knees, but a relationship between decreasing medial tibial slopes for increasing varus angle was noted, which may be consequence of the larger loads that pass through the medial condyle in more varus knees. The high medial loads and the larger extension facet in the medial condyle articulating against the medial tibial plateau could result in a flattening of the surface, thus reducing the slope. In addition, in implanted knees, computational studies have also shown contact stresses concentrated in smaller regions along with an increase in ligament stresses with flatter slopes [22]. This would imply that in severely varus knees, flatter slopes may lead to higher and more localized stresses. For varus tibiae, the posterior slope needs to be resolved carefully, as there is evidence in the literature of further varus alignment if the posterior slope is increased and is externally rotated [32].

A limitation of the study is that the varus group had OA and the normal group was asymptomatic. However, the OA in the varus group was confined to the anterior of the medial tibio-femoral articulation only, and none of the patients were candidates for TKR. Previous work comparing varus knees to normal has had the same limitation [8, 10, 24, 27, 30], but even asymptomatic varus patients might have cartilage wear in the anterior of the medial tibio-femoral articulation that could influence measurement of axes [14]. Another drawback is that the relative positioning of femoral and tibial axes could not be measured due to lack of information on the supine alignment (flexion angle) during the CT scanning of the patients. If included, the relative movement of the femur on the tibia could have also been studied and kinematic differences between the two groups investigated. Complete patient demographics, BMI, height and age were also not available, and the results might not apply for ethnicity other than the Caucasian population studied.

The clinical implication of the study is related to implant alignment and the design of patient-specific instrumentation and implants in knee arthroplasty. Reference axes were identified (based on the femoral neck and tibial tubercle) that are significantly different between normal and varus knees. Caution should therefore be exercised using these axes for implant alignment in varus knees, whether manually performed during surgery or by preoperative planning for patient-specific guides. However, specific axes were also identified that are not different in normal and varus knees. In the design of patient-specific knee replacement, or even varus specific knee replacement, the data from the study can also help inform implant design by quantifying the larger medial extension facet and the anterior translation of a sphere used to characterize this surface from its posterior to anterior boundary. Another, secondary, clinical implication of the study was that the coronal tibial slope could be measured using the deepest points of the troughs of the tibial plateaus which may be useful when osteophytes make the traditional method of measuring the slope difficult.

## Conclusions

The study found that a varus knee is significantly different to a normal knee in terms of femoral neck version, position of the tibial tubercle and morphology of the medial femoral and tibial condyle. These findings should be considered when selecting alignment axes, or designing implants or instrumentation for knees with a varus deformity.
